# Stereotactic body radiotherapy optimization to reduce the risk of carotid blowout syndrome using normal tissue complication probability objectives

**DOI:** 10.1002/acm2.13563

**Published:** 2022-02-23

**Authors:** Gregory Szalkowski, Zeynep Karakas, Mustafa Cengiz, Eric Schreiber, Shiva Das, Gozde Yazici, Gokhan Ozyigit, Panayiotis Mavroidis

**Affiliations:** ^1^ Department of Radiation Oncology University of North Carolina North Carolina Chapel Hill USA; ^2^ Faculty of Medicine Department of Radiation Oncology Hacettepe University Sihhiye Ankara Turkey

**Keywords:** carotid blowout syndrome, LKB, logit, NTCP, radiobiological parameters, relative seriality, SBRT

## Abstract

**Purpose:**

To determine the possibility of further improving clinical stereotactic body radiotherapy (SBRT) plans using normal tissue complication probability (NTCP) objectives in order to minimize the risk for carotid blowout syndrome (CBOS).

**Methods:**

10 patients with inoperable locally recurrent head and neck cancer, who underwent SBRT using CyberKnife were analyzed. For each patient, three treatment plans were examined: (1) cone‐based without delineation of the ipsilateral internal carotid (clinical plan used to treat the patients); (2) cone‐based with the carotid retrospectively delineated and spared; and (3) Iris‐based with carotid sparing. The dose–volume histograms of the target and primary organs at risk were calculated. The three sets of plans were compared based on dosimetric and TCP/NTCP (tumor control and normal tissue complication probabilities) metrics. For the NTCP values of carotid, the relative seriality model was used with the following parameters: *D*_50_ = 40 Gy, γ = 0.75, and *s* = 1.0.

**Results:**

Across the 10 patient plans, the average TCP did not significantly change when the plans were re‐optimized to spare the carotid. The estimated risk of CBOS was significantly decreased in the re‐optimized plans, by 14.9% ± 7.4% for the cone‐based plans and 17.7% ± 7.1% for the iris‐based plans (*p* = 0.002 for both). The iris‐based plans had significant (*p* = 0.02) reduced CBOS risk and delivery time (20.1% ± 7.4% time reduction, *p* = 0.002) compared to the cone‐based plans.

**Conclusion:**

A significant improvement in the quality of the clinical plans could be achieved through the delineation of the internal carotids and the use of more modern treatment delivery modalities. In this way, for the same target coverage, a significant reduction in the risk of CBOS could be achieved. The range of risk reduction varied depending on the proximity of carotid artery to the target.

## 1 INTRODUCTION

Head and neck cancer accounts for approximately 5% of the new cancer cases worldwide, corresponding to about 650,000 cases.^1^ Of these, approximately two‐thirds will present as locally advanced, of which an estimated 3%–50% will report local recurrence or persistent disease.^2^, ^3^, ^4^, ^5^, ^6^ While some of these patients can receive surgery, the large majority will require additional local treatment due to the high rate of recurrence.^7^, ^8^, ^9^, ^10^, ^11^ However, while re‐irradiation has been found to increase local control and overall survival, it does also increase the probability and severity of normal tissue toxicity.^12^, ^13^, ^14^

Stereotactic body radiotherapy (SBRT) can be used to help lower these risks, as the sharp dose gradients used in SBRT allow for greater sparing of organs at risk in close proximity to the target. CyberKnife (CK, Accuray, Sunnyvale, CA) robotic radiosurgery can provide even greater sparing, as the non‐isocentricity of the delivery allows for sharper gradients than can be achieved using standard linear accelerators, and the built‐in intra‐fraction imaging allows for the use of smaller treatment margins. The CK system has three distinct collimation methods that can be used for delivery: fixed cones, Iris collimator, and most recently a multi‐leaf collimator (MLC). While the use of the fixed cone collimator restricts the planner to only three aperture sizes for a single treatment, the Iris collimator allows for up to 12 field sizes to be used in a single treatment, allowing for greater flexibility in the plan design.^15^ Recent studies have shown that SBRT results in a higher local control rate compared to IMRT, but the rate of occurrence of carotid blowout syndrome (CBOS) also increased from 5% for IMRT to 17.3% for SBRT.^16^, ^17^

CBOS, which is a rupture of the carotid artery, has high associated neurologic morbidity and mortality rates, 40% and 60%, respectively.^18^ The purpose of this study is to utilize normal tissue complication probability (NTCP) and tumor control probability (TCP) models developed for SBRT re‐irradiation of recurrent head and neck cancer^19^ to guide plan optimization to lower the risk of this devastating toxicity while maintaining tumor control. Further, we investigate whether the use of the Iris collimator allows for the creation of higher quality plans compared to the fixed collimator.

## 2 METHODS

### 2.1 Patient selection and dose prescription

The patient data used for this study consist of 10 patients treated with SBRT at the Department of Radiation Oncology, Hacettepe University, Ankara, Turkey using CK (Accuray, Sunnyvale, CA) with complete treatment and follow‐up records. These patients are a subset of the 61 patients that were used to derive the NTCP model parameters.^19^ These patients had recurrent, unresectable, and previously irradiated head and neck cancer, and their recurrence was confirmed either radiologically or histopathologically. Informed consent was obtained from all patients prior to re‐irradiation. All patients were immobilized using a thermoplastic mask that was used for their imaging, CT and magnetic resonance imaging (MRI), and for treatment. The CT and MRI images were rigidly registered prior to contouring. As the delineation of the internal carotid was not standard clinical practice at the time of the patient's initial treatment, it was retrospectively contoured for the purpose of these studies.

**FIGURE 1:**
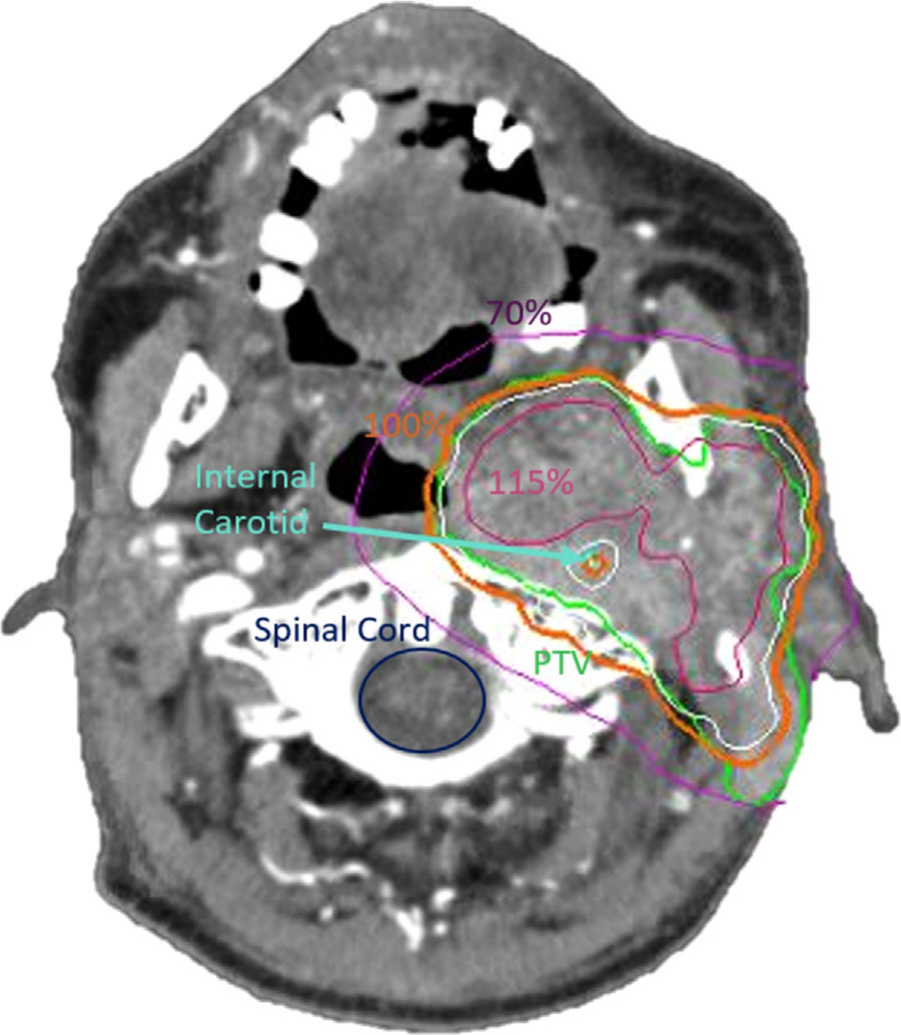
A slice from a representative treatment plan, showing the target, organs at risk and isodose lines. The prescription dose for this patient was 30Gy in five fractions

This subset of patients received a median prescribed dose of 66.0 Gy (range, 60.0–70.0 Gy), delivered in fractional doses of 1.8–2.0 Gy. For the SBRT re‐irradiation, one patient was treated with three fractions to 30.0 Gy, one patient was treated with five fractions to 35.0 Gy, and the remaining eight patients were treated with five fractions to 30.0 Gy. Figure 1 shows a representative clinical plan, including the internal carotid contour and isodose lines. The median tumor volume was 52.2 cm^3^ (range, 22.4–166.5 cm^3^). Patients were scheduled for follow‐up visits monthly for the first 3 months following the SBRT treatment, with the 4th visit scheduled on the 6‐month post‐RT. Patients then had a follow‐up visit every 6 month thereafter. Of the 10 patients, two ultimately suffered from the CBOS post‐treatment. Table 1 shows a summary of the characteristic for all of the patients included in this study. For each of those 10 patients, three treatment plans were examined: (1) cone‐based without delineation of the ipsilateral internal carotid (clinical plan used to treat the patients); (2) cone‐based with the carotid retrospectively delineated and spared; and (3) Iris‐based with carotid sparing.

**TABLE 1:** Treatment characteristics for patients with and without CBOS

**Diagnosis**	**Treatment**	**GTV volume (cm^3^)**	**Age (y)**	**Gender**	**Primary RT (Gy)**	**Fractions (#)**	**CK dose (Gy)**	**Dmax (tumor) (Gy)**	**Dmax (carotid) (Gy)**	**CBOS**
Larynx	Every other day	127.5	15	w	70	5	30	37.5	33.8	Present
Oral cavity	Every other day	51.4	58	m	70	5	30	38.5	36.9	Present
Larynx	Continuous	32.9	59	m	62	5	30	37.5	30.7	Absent
Oral cavity	Every other day	166.5	67	m	63	5	30	38.5	37.8	Absent
Hypopharynx	Continuous	54.4	54	m	70	3	30	42.9	32.9	Absent
Hypopharynx	Continuous	48.3	19	m	66	5	35	41.2	23.4	Absent
Paranasal sinus	Every other day	53.1	60	w	64	5	30	40.0	32.2	Absent
Paranasal sinus	Continuous	27.4	53	w	60	5	30	35.3	26.6	Absent
Nasopharynx	Continuous	170.4	48	m	69	5	30	41.7	38.8	Absent
Nasopharynx	Continuous	22.4	40	w	66	5	30	42.9	38.6	Absent

### 2.2 Radiobiological quantities

The doses in the DVHs were converted to equivalent doses of 2 Gy per fraction (EDQ_2Gy_) based on the linear quadratic model.^20^, ^21^ CBOS is considered to be a late complication. For this reason, an α/β value of 3 Gy was used to account for the fractionation effects of the physical dose. The relative seriality (RS) and Lyman–Kutcher–Burman (LKB) NTCP models were used to estimate the risks of CBOS for the different dose distributions to internal carotid.^22^, ^23^ From the NTCP values, the biologically effective uniform dose ($\bar{\bar{D}}$) can be derived. The basic parameters of the NTCP models are: *D*_50_ for RS (or TD_50_ for LKB), which is the dose for a complication rate of 50%, γ for RS (or m for LKB), the slope (gradient) of the dose–response curve and *s* for RS (or *n* for LKB), the parameter that accounts for the volume dependence of the organ. These parameters were derived from the complete patient cohort of 61 patient in an earlier study.^19^ Regarding the parameters describing volume dependence, it should indicated that an *s* value close to 1 indicates organs of serial behavior, which is associated with the maximum dose (*D*_max_) to the organ.

### 2.3 Fixed and Iris based re‐planning using NTCP objectives

All of the re‐optimized plans were created using the precision treatment planning system (Accuray, Sunnyvale, CA) and the VOLO optimizer. Based on the work from Mavroidis et al.,^19^ the risk of CBOS is greatly reduced if the max dose to the carotid is reduced below 34 Gy, so this was added as an optimization objective in addition to the standard PTV coverage and other organ at risk (OAR) sparing objectives. That work also indicated that the risk of CBOS is decreased if the full circumference receives less than 30 Gy. However, this does not translate itself easily to an optimization objective, so a secondary optimization goal of *D*_max_ < 30 Gy was added as well as a surrogate. Coverage of the gross tumor volume (GTV), and by extension maintaining the TCP, was set as the primary goal. After optimization, the dose volume histograms (DVHs) for the relevant structures; GTV, carotid, spinal cord, optic nerves, and brainstem, were exported and analyzed using our TCP/NTCP model to assess the plan quality.

## 3 RESULTS

**FIGURE 2:**
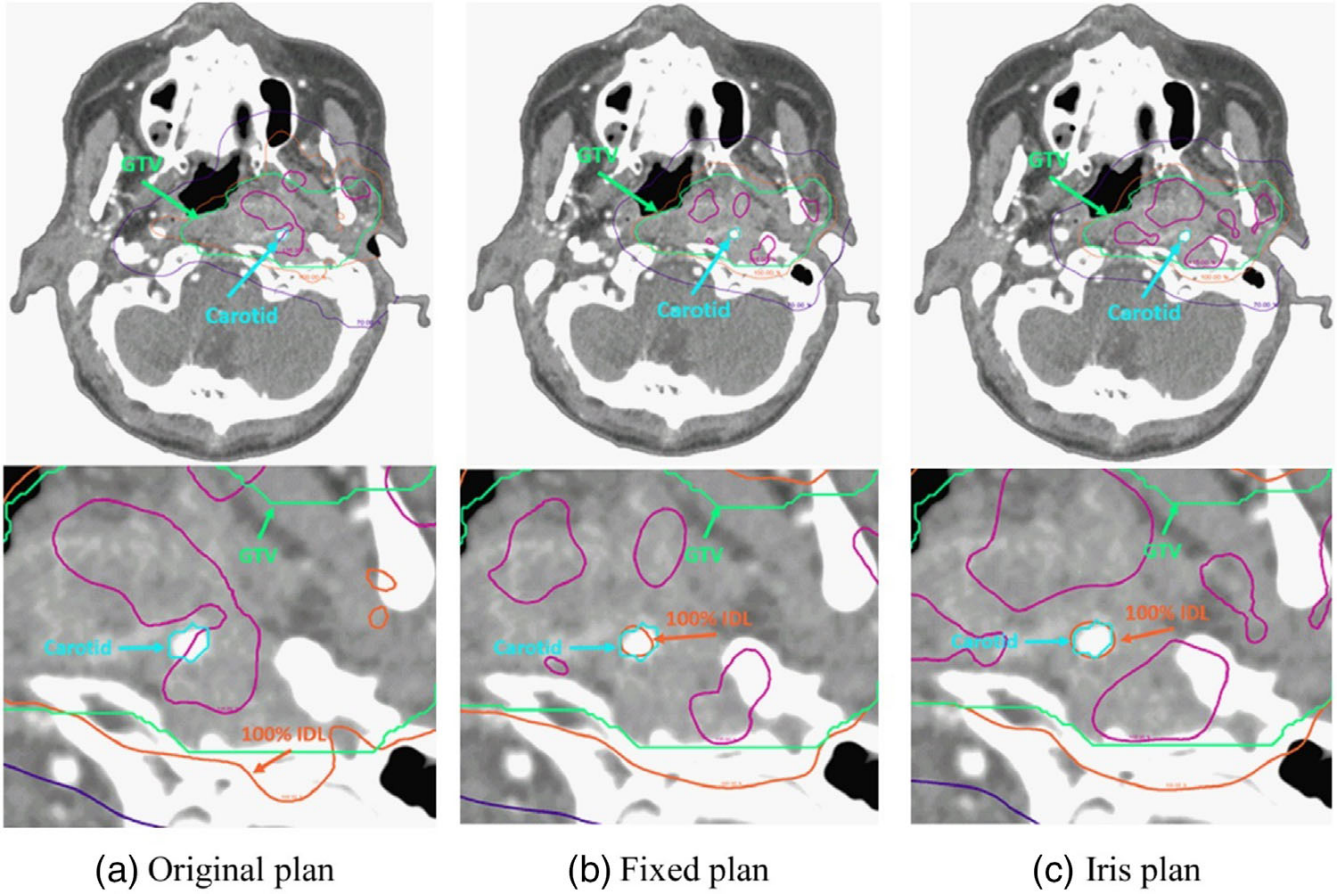
Comparison of the original plan (a) to the re‐optimized fixed cone (b) and Iris (c) plans, for the whole slice (top row) and focused on the carotid (bottom row). In the re‐optimized plans, the 100% isodose line (orange) is carved around the carotid (teal) and the 115% (light purple) is pushed away

**FIGURE A1:**
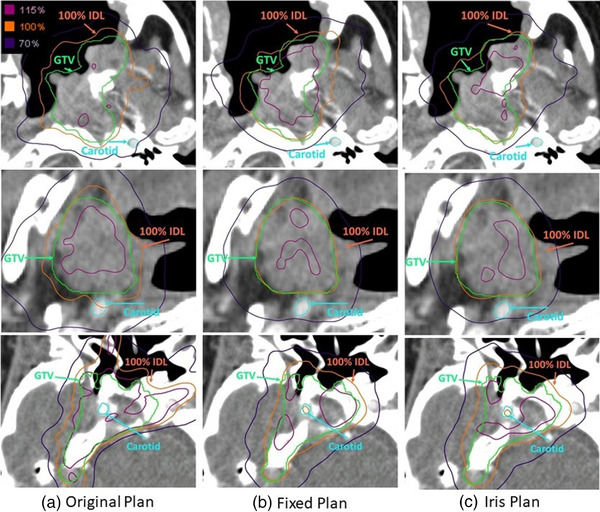
Further comparison of the original plans (a) to the re‐optimized fixed cone (b) and Iris (c) plans, for plans, where the carotid is distant (top row), nearby (middle row), or overlaps (bottom row) the GTV. As in the previous example, dose is pushed away from the carotid in the re‐optimized plans, even when the carotid overlaps the GTV

The re‐optimized plans were able to decrease the dose to the carotid with typically small changes to the GTV and other OAR doses. Figure 2 shows how the re‐optimized plans were able to carve out a region of lower dose around the carotid in cases, where the carotid passes through the GTV trying to keep this lower dose region within the prescribed isodose line. (See Figure A1 in the Appendix for further examples.) In cases where more dose was pushed into other OARs to reduce dose to the carotid, the plans still met the clinical goals for those structures. Table 2 shows a comparison of the dose to the GTV, carotid, and other relevant OARs across the different plans (Table A1 shows this comparison for each patient).

**TABLE 2:** Comparison of dosimetric indices such as: average dose (*D*_mean_), maximum dose (*D*_max_), and minimum dose (*D*_min_) of the different plans. For the clinical plans the absolute doses are shown, whereas for the re‐optimized plans, their differences from the clinical plans are presented

		*D*_mean_(Gy)			*D*_max_ (Gy)			D_min_ (Gy)		
Structure	Volume (cm^3^)	Original	Cones (Δ)	Iris (Δ)	Original	Cones (Δ)	Iris (Δ)	Original	Cones(Δ)	Iris(Δ)
GTV	64.7 ± 49.3	34.3 ± 1.9	0.0 ± 1.5	0.4 ± 1.1	39.6 ± 2.0	−0.1 ± 2.5	−0.5 ± 2.7	25.2 ± 4.4	−0.4 ± 2.8	0.3 ± 2.5
Carotid	2.4 ± 1.3	18.9 ± 5.3	−3.7 ± 2.1	−4.9 ± 2.6	33.3 ± 5.2	−7.0 ± 3.6	−8.1 ± 4.6	1.7 ± 2.1	1.3 ± 3.5	1.0 ± 2.9
Spinal cord	15.3 ± 13.4	3.3 ± 1.8	1.2 ± 1.2	1.7 ± 1.7	9.9 ± 5.2	0.7 ± 3.6	0.9 ± 1.8	0.7 ± 1.3	0.4 ± 0.7	0.6 ± 0.5
Optic chiasm	1.3 ± 1.2	6.5 ± 3.4	1.3 ± 2.3	1.5 ± 2.4	16.4 ± 7.0	−1.5 ± 4.1	−1.6 ± 3.9	2.4 ± 1.2	2.0 ± 2.6	1.4 ± 2.6
Brain stem	30.7 ± 8.7	5.0 ± 2.7	1.1 ± 2.8	0.5 ± 2.9	15.6 ± 8.0	1.1 ± 5.4	1.6 ± 5.4	0.6 ± 0.6	0.8 ± 0.9	0.6 ± 0.6

Using our NTCP model, both the cones and the Iris re‐optimized plans were found to significantly (*p* = 0.002 for both) decrease the estimated risk of CBOS while maintaining similar TCP (*p* = 0.49) compared to the original plans using the Wilcoxon rank‐sum test. The estimated NTCP reduction from the original plans for the carotid was 14.9% ± 6.6% for the cones plans and 17.7% ± 7.1% for the Iris plans. Additionally, utilizing the Iris collimator was found to significantly (*p* = 0.02) decrease the estimated risk of CBOS compared the plans utilizing the cones collimator. Table 3 shows a comparison of the TCP and the NTCP for the carotid for the original, fixed cones, and Iris plans for the patients investigated in this study.

**TABLE 3:** Comparison of the average radiobiological indices such as: tumor control probability (TCP), normal tissue complication probability (NTCP) and biologically effective uniform dose (BEUD) of the different plans. For the clinical plans the absolute probabilities or biological doses are shown, whereas for the re‐optimized plans, their differences (Δ) from the clinical plans are presented

	TCP (%)	BEUD (Gy)	NTCP carotid (%)	BEUD (Gy)
Original	82.1% ± 8.0%	46.7 ± 3.6	42.4% ± 25.8%	36.7 ± 14.0
Cones (Δ)	0.9% ± 5.9%	0.13 ± 1.8	−14.9% ± 7.4%	−9.7 ± 3.2
Iris (Δ)	1.9% ± 4.6%	0.32 ± 1.4	−17.7% ± 7.1%	−11.8 ± 3.8

**FIGURE 3:**
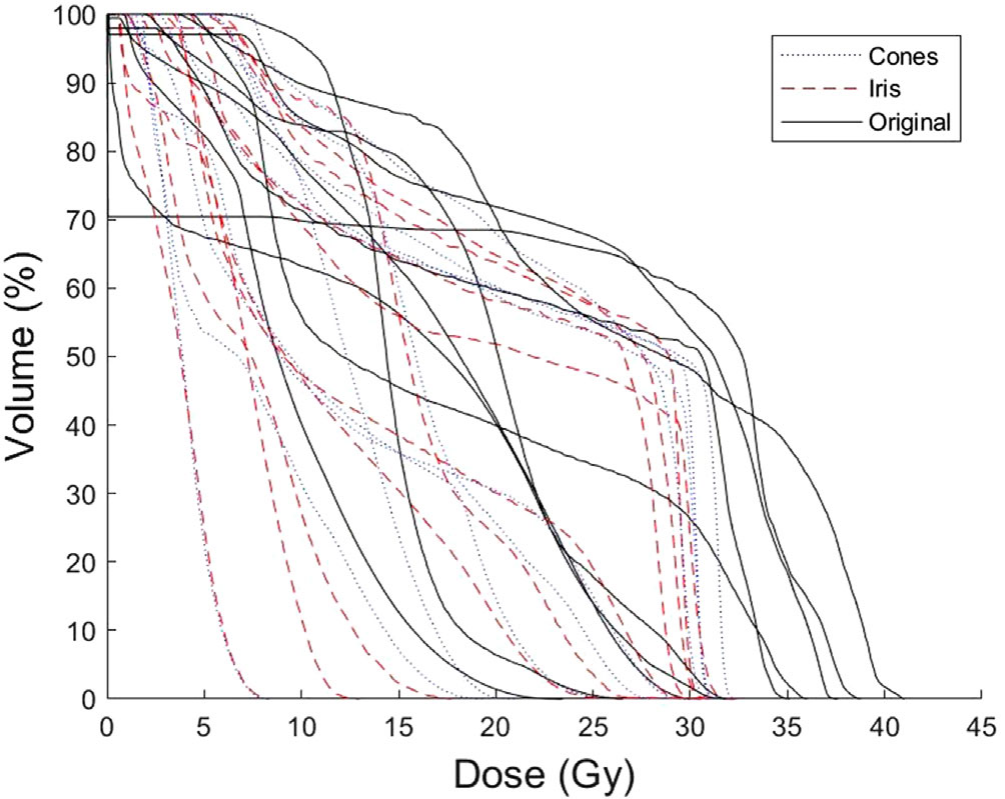
Comparison of the carotid dose–volume histograms for the original (solid), fixed cones (dotted), and Iris (dashed) plans, with each line corresponding to a single plan. The re‐optimized plan sharply reduced the volume above 32 Gy and generally reduced the mean dose to the carotid

Figure 3 shows a comparison of the carotid DVHs across the different plans. Generally, optimizing off of the carotid led to a lower overall dose, though in some cases, only the maximum dose could be meaningfully reduced without compromising coverage, resulting in DVHs with a sharp drop‐off at 34 Gy.

In terms of treatment efficiency, the Iris plans were significantly faster (*p* = 0.002), taking on average 20.1% ± 7.4% less time to deliver, corresponding to about 8 min. This does not include the further time savings of not needing the therapists to enter the room to manually change the collimator.

## 4 DISCUSSION

The comparison of treatment plans according to optimization of carotid artery doses showed that it is possible to meaningfully reduce the risk of CBOS (*p* = 0.002) while maintaining tumor control (*p* = 0.49) for patients receiving CK based SBRT for treatment of recurrent head and neck cancers. Additionally, the newer Iris collimator for the CK allows for further sparing of the carotid while also giving a large reduction in the treatment time (*p* = 0.002).

It is interesting to note that, from the results shown inTable A2, for 5 of the 10 patients, the re‐optimized plans were able to both reduce the risk of CBOS while increasing the tumor dose, even for the plans that used the same fixed cone collimator as the original plans. It was found that that this is mainly due to the use of the VOLO optimizer in this work, as opposed to the previous sequential optimizer used to create the original plans, as the VOLO optimizer has been shown to increase plan quality.^24^

This study includes several limitations. First, it is a retrospective study based on the re‐planning of previously treated patients. While our model indicates that dose reduction could be achievable in the carotid artery, which would lead to a lower incidence of CBOS while maintaining tumor control rates, this has not been shown clinically in this study, though it has been demonstrated elsewhere in the literature.^14^ Additionally, this study only looks at the data from 10 subjects, for whom their anatomy may be favorable for optimizing off of the carotid, and it is possible that those results may not be representative of other patient cohorts with different anatomical characteristics. Finally, for cases where the carotid passes through the target, changes in internal anatomy may result in the carotid moving out of the area spared in the plan and into a higher dose region. Due to the limited imaging available on the CK unit, it may be difficult to verify the carotid positioning even with contrast, so a planning risk volume may be prudent.

## 5 CONCLUSION

Overall, the NTCP‐guided optimization was able to produce plans with a lower estimated risk of CBOS than plans produced without this optimization. Further, the use of the Iris collimator provided a significant benefit, both in terms of plan quality, as assessed by the NTCP and TCP metrics, and in terms of delivery time. Future work could investigate the impact of using the newer MLC collimator that was introduced with the M6 series CK units.

## CONFLICT OF INTEREST

The authors declare that there is no conflict of interest that could be perceived as prejudicing the impartiality of the research reported.

## AUTHOR CONTRIBUTIONS

Data collection: Mustafa Cengiz, Gozde Yazici, Gokhan Ozyigit. Data processing: Gregory Szalkowski, Zeynep Karakas, Panayiotis Mavroidis. Data analysis/statistics: Gregory Szalkowski, Zeynep Karakas, Eric Schreiber, Panayiotis Mavroidis. Manuscript writing: Gregory Szalkowski, Shiva Das, Mustafa Cengiz, Panayiotis Mavroidis.

## 1

**TABLE A1:** Comparison of dosimetric indices such as: average dose (*D*_mean_), maximum dose (*D*_max_) and minimum dose (*D*_min_) of the different plans. For the clinical plans the absolute doses are shown, whereas for the re‐optimized plans, their differences (Δ) from the clinical plans are presented

		***D*_mean_ (Gy)**			***D*_max_ (Gy)**			***D*_min_ (Gy)**		
**Structure**	**Volume (cm^3^)**	**Original**	**Cones (Δ)**	**Iris (Δ)**	**Original**	**Cones (Δ)**	**Iris (Δ)**	**Original**	**Cones (Δ)**	**Iris (Δ)**
	Patient 1									
GTV	7.4	38.6	−0.6	−0.4	41.2	−1.3	−1.0	34.6	−11.5	−1.4
Carotid	2.0	9.3	−0.5	−5.7	23.7	−15.3	−15.5	0.9	−11.6	−2.4
Spinal Cord	10.5	2.2	−0.6	−0.6	6.8	−2.0	−2.1	0.2	−0.1	−0.1
	Patient 2									
GTV	52.7	33.1	−0.3	−0.7	42.9	−4.9	−6.7	22.2	+1.4	+1.6
Carotid	3.1	14.1	−2.8	−3.6	32.2	−2.2	−1.6	0.1	+0.7	+0.5
Spinal cord	44.3	1.7	+2.1	+1.5	5.4	3.4	3.3	0.0	+0.5	+0.5
	Patient 3									
GTV	163.8	33.2	+1.0	+1.4	38.5	+1.7	+1.1	22.3	‐2.1	+0.8
Carotid	3.3	26.0	−2.8	−3.8	37.7	−6.4	−6.7	0.0	+7.4	+4.8
Spinal cord	6.42	6.8	+2.6	+5.0	18.7	+2.7	+1.3	3.7	−0.7	+0.5
Optic chiasm	0.56	11.5	+0.7	+1.6	23.3	−5.6	−7.4	3.0	+6.4	+6.1
	Patient 4									
GTV	26.81	32.4	+3.14	+1.5	37.3	+5.1	+1.4	23.7	−2.0	−0.7
Carotid	0.88	14.7	−7.4	−7.5	26.5	−7.3	−8.8	6.0	−4.2	−4.9
Spinal cord	5.34	1.7	+1.7	+0.8	5.4	+5.3	+2.7	0.4	+0.5	0.0
Optic chiasm	0.61	5.4	−0.9	+1.2	17.3	−4.8	−3.3	1.3	+0.7	+0.6
Brain stem	19.61	5.7	−0.3	−1.3	18.6	0.0	−2.9	0.7	+2.3	+0.2
	Patient 5									
GTV	31.5	33.5	+0.4	+0.8	37.5	−0.1	0.0	27.5	+1.0	+0.9
Carotid	5.07	16.7	−4.4	−9.5	29.9	−8.9	−17.0	0.0	+5.5	+2.8
Spinal cord	8.46	3.3	+1.6	+1.4	8.2	0.0	0.0	0.0	+1.1	+1.1
	Patient 6									
GTV	21.71	36.6	−2.5	−1.8	42.9	−2.4	−1.9	17.4	+6.6	+6.2
Carotid	0.85	26.9	−5.9	−7.5	41.0	−9.6	−8.8	3.6	0.0	+0.7
Optic chiasm	0.4	8.0	5.8	5.9	20.3	+1.9	+2.7	3.3	+3.8	+3.4
Brain stem	27.18	4.0	+6.7	+6.6	17.6	+11.2	+12.4	0.7	+2.4	+1.4
	Patient 7									
GTV	51.25	33.7	+0.8	+0.5	40.0	0.0	−0.4	23.8	−2.8	−2.5
Carotid	1.23	18.8	−2.7	−2.8	32.0	−3.9	−4.7	0.0	+1.5	+1.8
Optic chiasm	0.68	4.9	+1.6	+1.4	17.8	−6.1	−5.0	2.8	+1.4	+0.4
Brain stem	24.84	3.7	+0.1	−0.3	9.3	−1.2	−1.4	0.9	+0.2	+0.2
	Patient 8									
GTV	49.96	34.7	−0.8	+0.7	38.4	+0.1	−2.7	28.7	−2.0	−1.1
Carotid	2.49	17.8	−4.6	−0.1	36.0	−6.3	−6.0	4.3	−3.1	−0.9
Brain stem	46.68	2.3	+1.3	0.0	0.5	+3.5	+3.5	0.0	+0.7	+0.8
	Patient 9									
GTV	123.6	32.5	+0.4	0.0	37.4	0.0	+3.6	26.6	−3.5	−2.9
Carotid	3.65	21.9	−0.5	0.0	35.2	−2.7	−4.2	1.8	+0.7	+0.4
Spinal Cord	16.55	4.2	−3.0	0.0	15.2	−5.6	0.0	0.0	+1.3	+1.4
Optic Chiasm	1.66	0.5	+2.0	0.0	1.5	+3.4	+2.0	0.4	+1.2	+0.4
Brainstem	29.85	3.7	−2.4	0.0	12.5	−6.4	−2.9	1.0	−0.3	−0.2
	Patient 10									
GTV	117.87	34.8	−1.5	−1.8	39.8	0.0	+1.3	24.7	+0.3	+1.8
Carotid	1.6	23.0	−1.0	−0.8	38.7	−7.6	−7.7	0.0	+5.0	+5.7
Optic chiasm	3.73	8.5	−1.2	−2.5	18.3	+2.5	+1.7	3.7	−1.9	−2.2
Brain stem	35.82	10.7	+1.1	−1.0	30.3	−0.5	+0.8	0.0	+1.6	+1.5

**TABLE A2:** Comparison of radiobiological indices such as: tumor control probability (TCP), normal tissue complication probability (NTCP), and biologically effective uniform dose (BEUD) of the different plans. For the clinical plans the absolute probabilities or biological doses are shown, whereas for the re‐optimized plans, their differences (Δ) from the clinical plans are presented

	TCP (%)	BEUD (Gy)	NTCP carotid (%)	BEUD (Gy)
	Patient 1			
Original	97.9%	56.3	3.3%	13.5
Cones (Δ)	−0.6%	−0.9	−3.1%	−10.3
Iris (Δ)	−0.4%	−1.1	−3.1%	−10.2
	Patient 2			
Original	70.4	43.2	25.3%	28.6
Cones (Δ)	2.7%	0.5	−15.2%	−8.5
Iris (Δ)	1.7%	0.3	−17.0%	−9.9
	Patient 3			
Original	78.1%	44.8	72.1%	52.5
Cones (Δ)	4.6%	1.2	−18.4%	−10.7
Iris (Δ)	6.6%	1.9	−20.9%	−11.9
	Patient 4			
Original	73.9%	43.9	9.2%	19.5
Cones (Δ)	12.7%	3.5	−7.3%	−8.4
Iris (Δ)	8.0%	2.0	−7.9%	−9.9
	Patient 5			
Original	83.0%	46.2	25.4%	28.7
Cones (Δ)	1.2%	0.4	−20.3%	−12.9
Iris (Δ)	2.9%	1.0	−24.7%	−21.3
	Patient 6			
Original	87.6%	47.8	75.5%	55.0
Cones (Δ)	−5.9%	−2.0	−23.0%	−13.8
Iris (Δ)	−2.4%	−0.9	−27.4%	−15.9
	Patient 7			
Original	79.9%	45.3	32.2%	31.8
Cones (Δ)	−0.1%	0.0	−13.6%	−6.6
Iris (Δ)	−1.9%	−0.5	−15.7%	−7.7
	Patient 8			
Original	87.9%	47.9	45.5%	37.9
Cones (Δ)	3.1%	1.5	−25.2%	−11.7
Iris (Δ)	−2.2%	−0.8	−23.6%	−10.9
	Patient 9			
Original	74.0%	43.9	61.8%	46.1
Cones (Δ)	1.9%	0.4	−4.1%	−2.2
Iris (Δ)	10.3%	2.7	−16.5%	−8.3
	Patient 10			
Original	88.1%	48.0	73.6%	53.6
Cones (Δ)	−10.7%	−3.3	−19.3%	−11.4
Iris (Δ)	−3.2%	−1.2	−20.5%	−12.0
